# Ethanol in herbal medicinal products for children: Data from pediatric studies and pharmacovigilance programs

**DOI:** 10.1007/s10354-016-0474-x

**Published:** 2016-07-28

**Authors:** Olaf Kelber, Barbara Steinhoff, Christian Nauert, Andreas Biller, Martin Adler, Heba Abdel-Aziz, Samuel N. Okpanyi, Karen Kraft, Karin Nieber

**Affiliations:** 1 Board of the Corporative Members, Gesellschaft für Phytotherapie e. V. (GPT), Cologne, Germany; 2 Innovation & Development, Phytomedicines Supply and Development Center, Bayer Consumer Health, Steigerwald Arzneimittelwerk GmbH, Havelstr. 5, 64295 Darmstadt, Germany; 3 Bundesverband der Arzneimittel-Hersteller, Bonn, Germany; 4 Cassella-med GmbH & Co. KG, Cologne, Germany; 5 Dr. Loges + Co. GmbH, Winsen, Germany; 6 Institute for Integrative Medicine Siegen, Siegen, Germany; 7 Medical Affairs, Phytomedicines Supply and Development Center , Bayer Consumer Health, Steigerwald Arzneimittelwerk GmbH , Darmstadt, Germany; 8 Chair of Complementary Medicine, Center of Internal Medicine, Universitätsmedizin Rostock, Rostock, Germany; 9 Scientific Board, Gesellschaft für Phytotherapie e. V. (GPT), Cologne, Germany; 10 Institute of Pharmacy, University of Leipzig, Leipzig, Germany

**Keywords:** Pediatric medicinal product, Phytotherapy, Liquid dosing form, Labeling, Benefit–risk assessment, Kinderarzneimittel, Phytotherapie, Flüssige Darreichungsform, Fach- und Gebrauchsinformation, Nutzen-Risiko-Bewertung

## Abstract

Herbal medicinal products are indispensable in children, e. g., in functional gastrointestinal diseases and coughs and colds, especially when available in liquid dosing forms for which dosing can be adapted ideally to different age groups. Despite being generally accepted as safe, the ethanol content of many of these products, necessary for Galenic reasons, has raised questions regarding their safety. Therefore, safety data from more than 50,000 children in noninterventional pediatric studies with these products, as well as data from routine clinical use in several million children, were assessed. No evidence of the involvement of the ethanol content in any adverse drug reactions was found. This allows us to conclude that these herbal medicinal products are safe in the age groups for which they are authorized or registered and that the present labeling is adequate to allow for their safe use in the pediatric population.

## Zusammenfassung

Pflanzliche Arzneimittel gehören bei Kindern zum unverzichtbaren Arzneimittelschatz, beispielsweise bei funktionellen gastrointestinalen Erkrankungen sowie Husten und Erkältung. Dies trifft insbesondere auf flüssige Darreichungsformen zu, bei denen die Dosierung ideal an unterschiedliche Altersgruppen angepasst werden kann. Obwohl sie generell als sehr sicher gelten, wirft der Alkoholgehalt vieler dieser Arzneimittel, der aus galenischen Gründen notwendig ist, immer wieder Fragen auf. Daher wurden Sicherheitsdaten von über 50.000 Kindern aus nichtinterventionellen Studien (NIS) sowie aus der Routine-Pharmakovigilanz bei Arzneimitteln, die an mehreren Millionen Kindern angewendeten wurden, ausgewertet. Es wurden keine Hinweise auf durch den Alkoholgehalt bedingte Nebenwirkungen gefunden. Daraus lässt sich schließen, dass diese pflanzlichen Arzneimittel in allen Altersgruppen, für welche sie zugelassen oder registriert sind, gefahrlos angewendet werden können, und dass die gegenwärtigen Texte in Fach- und Gebrauchsinformationen eine unbedenkliche Anwendung bei Kindern sicherstellen.

## Introduction

Ethanol is a component of many herbal fluid preparations [1], since it is an excellent solvent for the extraction of herbal drugs and contributes to the stability of these medicines. Toxicological and pharmacokinetic evaluations have shown that the small amounts of ethanol applied with therapeutic doses are safe even in children [2, 3], while for potential surrogate solvents, e.g., glycerol or propylene glycol, toxicological proof of safety is not equally convincing [3, 4]. Typical doses of herbal medicines for children aged 6–12 years contain 0.07–0.18 g ethanol, which is equivalent to, e. g., 31–75 ml of apple juice (with an ethanol content of 0.3 %), and is eliminated from the blood within 1–3 min. These doses lead to a maximum blood ethanol content of 0.008–0.015 g/l, calculated under assumption of the worst case scenario.

Regulations in Germany [5] require a warning label if an adult single dose contains 0.5 g or more of ethanol and this also applies to lower doses of the same product for children. The French view [6] allows an ethanol content per single dose leading to a blood ethanol concentration not greater than 0.125 g/l. More recently, a draft for a Q & A paper of the European Medicines Agency (EMA) [7] was published. There, a warning is issued for a single dose of 6 mg/kg or more (this is equivalent to a dose of 120 mg ethanol or 50 ml apple juice in a 6-year-old with a body weight of 20 kg), stating that in children aged 6 years and younger, drowsiness, behavioral changes, and impaired ability to concentrate and participate in school activities can occur.

Given the different regulatory approaches, and against the background of public discussions triggered by the problem of recreational misuse of alcoholic beverages by adolescents [8–10], there is a need for a systematic evaluation of clinical and pharmacovigilance data on herbal medicines containing ethanol for children.

## Materials and methods

In the pharmacovigilance system in Germany, all reports of adverse drug reactions (ADRs) on herbal medicinal products in Germany are collected by the manufacturers. Line listings and data from periodic safety update reports (PSURs) on ADRs for herbal medicinal products up to 2009 containing ethanol were provided by manufacturers and assessed by the authors regarding a potential causality between the reports and the ethanol content of the medicines. For the same medicines, sales figures for Germany from IMS Health GmbH, Frankfurt/Main, Germany, were provided for the years 2005–2009, separately for total sales and sales prescribed by physicians and reimbursed by the health insurance. These Rx figures represent mainly prescriptions to children, as these medicines are reimbursed in Germany only for children up to 12 years of age and for adolescents up to 18 years of age with developmental disturbances (according to § 34, paragraph 1, No 1 and 2, Sozialgesetzbuch V, Germany) but they are not reimbursed in adults. Total sales figures (according to PSUR data) provided by the manufactures are given as daily doses (assuming adult doses for precautionary reasons) and patient numbers, assuming a treatment duration of 4 weeks.

In addition, all clinical studies in children available for these medicines were collected and the numbers of children and adults included as well as the ethanol doses corresponding to a single dose were listed. Studies available from congress abstracts or company reports are listed in Table 1. Single dose, daily dose, and ethanol content are according to information in the AMIS database accessible via DIMDI, Bonn, Germany. Ethanol content is given per single dose, as the small amounts of ethanol applied per single dose are metabolized within seconds or minutes so that dosing according to the therapeutic dosing schedule cannot lead to an accumulation [4]. Numbers of ADRs were listed and it was evaluated whether they were attributable to the ethanol content of the medicinal products.

**Table 1:** Studies with herbal medicinal products in children (in alphabetical order)

Herbal medicinal product	Number and age of children evaluated	Dose	% (v/v) ethanol	Gram ethanol per dose^b^	References	ADR^a^
Bronchicum® Elixir, oral liquid	*n* = 312 children (1–4 years)	2.5 ml 6 × daily	4.9	0.097	[14–16]	3
	*n* = 324 children (5–12 years)	5.0 ml 6 × daily		0.194		
Bronchicum® Elixir, oral liquid	*n* = 111 children (1–4 years)	2.5 ml 6 × daily	4.9	0.097	[16, 17]^d^	0
	*n* = 109 children (5–12 years)	5.0 ml 6 × daily		0.194		
Bronchicum® Tropfen, oral liquid	*n* = 110 school children (6–12 years )	25 drops 5 × daily	27.7^c^	0.274		0
Bronchicum® Elixir, oral liquid	*n* = 474 children (1–4 years)	2.5 ml 6 × daily	4.9	0.097	[18, 19]	3
	*n* = 365 children (5–12 years)	5.0 ml 6 × daily		0.194		
Bronchicum® Elixir, oral liquid	*n* = 200 infants (6–12 months)	1.0 ml 6 × daily	4.9	0.039	[20, 21]^d^	1
Contramutan® Tropfen, oral liquid	*n* = 85 infants (6–12 months)	2–3 drops 12 × daily	33.4^c^	0.040	[12]	0
	*n* = 95 children (1–6 years)	3–5 drops 12 × daily		0.066		
	*n* = 90 school children (6–12 years)	4–7 drops 12 × daily		0.092		
Contramutan® Saft, oral liquid	*n* = 174 infants (6–12 months)	1.5–2.0 ml 12 × daily	3.6	0.057		0
	*n* = 195 children (1–6 years)	2.0–3.5 ml 12 × daily		0.100		
	*n* = 188 school children (6–12 years)	3.0–5.0 ml 12 × daily		0.142		
Contramutan® Saft, oral liquid	*n* = 100 infants (6–12 months)	2.0 ml 12 × daily	3.6	0.057	[22]^d^	0
	*n* = 100 children (1–6 years)	4.0 ml 12 × daily		0.114		
	*n* = 100 school children (7–12 years)	5.0 ml 12 × daily		0.142		
Iberogast®, oral liquid	*n* = 642 infants (under 3 months)	0.33 ml 3 × daily	31.0^c^	0.081	[23]	0
	*n* = 7709 infants (3 months–3 years)	0.4 ml 3 × daily		0.098		
	*n* = 12,802 (4–6 years)	0.5 ml 3 × daily		0.122		
	*n* = 19,808 (7–12 years)	0.75 ml 3 × daily		0.184		
Iberogast®, oral liquid	*n* = 13 infants (under 3 months)	0.33 ml 3 × daily	31.0^c^	0.081	[24]	0
	*n* = 286 infants (3 month–3 years)	0.4 ml 3 × daily		0.098		
	*n* = 480 children (4–6 years)	0.5 ml 3 × daily		0.122		
	*n* = 1571 school children (7–12 years)	0.75 ml 3 × daily		0.184		
Iberogast®, oral liquid	*n* = 389 children (3–6 years)	0.5 ml 3 × daily	31.0^c^	0.122	[25]	4
	*n* = 591 school children (7–14 years)	0.75 ml 3 × daily		0.184		
Monpax® Saft, oral liquid	*n* = 118 infants (7–12 months) + infants (1–3 years)	2.5 ml 3 × daily	3.9	0.077	[26]	0
	*n* = 119 children (4–7 years)	2.5 ml 4 × daily		0.077		
	*n* = 113 school children (8–14 years)	5.0 ml 3 × daily		0.154		
Monapax® Tropfen, oral liquid	*n* = 118 infants (7–12 months) + infants (1–3years)	8 drops 3 × daily	32.8^c^	0.104		0
	*n* = 115 children (4–7 years)	10 drops 3 × daily		0.130		
	*n* = 115 school children (8–14 years)	10 drops 4 × daily		0.130		
Phytohustil® Hustenreizstiller Sirup, oral liquid	*n* = 100 children (under 3 years)	2.5–3 ml 4 × daily	1.1	0.026	[27]	0
	*n* = 115 children (3–6 years)	5 ml 4 × daily		0.043		
	*n* = 98 school children (6–12 years)	5 ml 5 × daily		0.043		
Phytohustil® Hustenreizstiller Sirup, oral liquid	*n* = 61 infants (under 3 months)	2.5 ml 4 × daily	1.1	0.022	[28]^d^	0
	*n* = 128 infants (3 month to 2 years)	3.4 ml 4 × daily		0.030		
	*n* = 188 children (3–5 years)	4.3 ml 4 × daily		0.037		
	*n* = 222 school children (6–12 years)	4.6 ml 5 × daily		0.040		
Phytobronchin® Saft S Lösung, oral liquid	*n* = 220 infants (under 1 year)	2.5ml 3 × daily	5.0–8.0	0.158	[29]	4
	*n* = 267 children (1–2 years)	3 m 3 ×daily		0.190		
	*n* = 352 children (3–5 years)	5 ml 3 × daily		0.316		
	*n* = 401 school children (6–12 years)	5 ml 3–4 × daily		0.316		
Soledum® Hustensaft, oral liquid	*n* = 18 infants (under 2 years)	1.25 ml 3–4 × daily	5.8	0.057	–	0
	*n* = 105 children (2–6 years)	2.5 ml 3–4 × daily		0.115		
	*n* = 19 school children (7–10 years)	5 ml 3–4 × daily		0.229		
*Sum*	*n = 50,316 children (0–12 years)*	–	–	–	–	*15*

^a^Total number of adverse drug reactions (ADRs): *n* = 15; all not severe and known; all unrelated to ethanol

^b ^For medicines applied as drops, a volume of 0.05 ml/drop was assumed

^c^Taken diluted with water, according to package leaflet

^d^References are congress abstracts or internal reports as part of a PSUR

## Results

### Data from clinical studies

For an evaluation of the experience gained from the therapeutic use of these medicines, 17 prospective and retrospective studies with ten herbal medicinal products were analyzed, containing ethanol at doses of 0.022–0.274 g per single application, depending on the age group. These studies cover 50,316 children of 0–12 years of age (Table 1). In these studies, altogether 15 ADRs have been described, none of which was attributable to the ethanol content of the medicines. The inclusion of three studies comprising 1041 children solely documented in congress abstracts or internal reports submitted to regulatory authorities does not change this picture.

### Pharmacovigilance data and patient exposure

Of the medicinal products listed in Table 1 and six additional medicinal products containing ethanol, during the period from 2005 to 2009 more than 764 million daily doses were sold, which corresponds to more than 33 million patients (Table 2).

**Table 2:** Safety data for some herbal medicinal products containing ethanol^a^

Herbal medicinal product	Period	Daily doses sold (*n*, million)	Corresponding number of patients (*n*, million)	ADRs attributable to ethanol
Allergo-loges®	2003–2009	3.4	0.1	None
Bronchicum® Elixir	2004–2009	19.3	1.6	None
Bronchicum® Tropfen	2004–2009	47.8	4.0	None
Contramutan® N Saft	2004–2009	4.8	0.4	None
Contramutan® Tropfen	2004–2009	2.1	0.2	None
Diarrhoesan®	2003–2009	4.4	0.6	None
Iberogast®	1993–2009	636.9	22.7	None
Influex®	1993–2009	15.5	1.5	None
Melrosum® Hustensirup	2004–2009	2.1	0.2	None
Monapax® Saft	2004–2009	7.2	0.6	None
Monapax® Tropfen	2004–2009	1.5	0.1	None
Phytobronchin® Saft S Lösung	1994–2009	3.4	0.4	None
Phytohustil® Hustenreizstiller Sirup	2000–2009	11.4	1.4	None
Soledum® Hustensaft	2004–2009	3.5	0.3	None
Soledum® Hustentropfen	2004–2009	1.3	0.1	None
*Total*		764.6	34.3	None

^a^Figures provided by the manufacturers. Figures are available partly from 1993 and partly from 2003/2004 on up to 2009

From the packages of these medicines sold in Germany between 2005 and 2009, 48.1 million were sold over the counter, of which 10.8 million prescriptions were covered by health insurance (Table 3). The latter can be assumed to have been used in children by about 90 %.

**Table 3:** Sales figures of some herbal medicinal products containing ethanol

Product	Sales figures total (million packages) 2005–9/2009	Sales figures Rx (million packages) 2005–9/2009
Bronchicum® Elixir	8.23	2.22
Bronchicum® Tropfen	5.81	0.58
Contramutan® Saft	3.16	1.52
Contramutan® Tropfen	0.90	0.18
Melrosum® Sirup	0.74	0.40
Monapax® Saft	2.69	1.51
Monapax® Tropfen	0.31	0.13
Soledum® Hustensaft	1.410	0.48
Soledum® Tropfen	0.17	0.05
Diarrhoesan®	1.04	0.58
Toxi-loges® Tropfen	0.77	0.02
Alergo-loges®	0.05	0.00
Iberogast®	20.63	2.86
Phytohustil® Hustenreizstiller Sirup	1.44	0.17
Phytobronchin® Saft S Lösung	0.41	0.07
Phytodolor®	0.29	0.09
*Sum*	*48.09*	*10.85*

ADRs in these medicines are covered by the pharmacovigilance system, as mentioned earlier, and no ADRs attributable to the ethanol content have been reported.

## Discussion

There is a wide use of herbal medicinal products containing small amounts of ethanol, in the range between 0.022 and 0.274 g per single dose, depending on the respective medicinal product and the different age groups. These doses are clearly within the range of ethanol doses given to children with normal food and drinks accepted as safe in this age group, for example, a typical serving of apple juice (100 ml) is expected to have 0.240 g ethanol [4]. Liquid dosage forms have an advantage for use in children, as it is possible to adapt doses to the respective age groups. Ethanol is specified as the extraction solvent for herbal medicinal products in all relevant pharmacopeias, and replacement by other solvents like glycerol or propylene glycol and by the addition of preservatives is not generally applicable, as the toxicological proof of safety for these substances is not equally convincing [4, 11]. Therefore these medicines are rated as safe for use in children by regulatory agencies and are also perceived as safe by health professionals and consumers [2]. The data presented here give strong support to these ratings.

The use of these medicines is very widespread: During the period 1993/2004–2009, more than 700 million daily doses were sold, which can be assumed to correspond to more than 30 million patients taking these medicinal products. Even when assuming that, for example, 50 % of the doses sold are not used (which is likely to be highly exaggerated in the case of OTC products paid by the patients themselves and indicated in frequently occurring diseases such as coughs and colds and functional gastrointestinal diseases), these figures are impressive, given that no ADRs attributable to ethanol have been reported.

According to Table 3, about 20 % of the 50 million packages of these medicinal products sold within this 5‑year period can be assumed to have been prescribed to children. Thus, about three million children (given very conservative assumptions probably significantly underestimating the real figures) can be assumed to be included in the overall patient figures given, underlining the relevance of the conclusions to children.

From studies, i. e., from therapeutic use controlled by physicians, the use of these products in more than 50,000 children of different age groups is documented. The studies differed in design (retro- and prospective noninterventional studies, NIS), which can be assumed to be the cause of the different figures of ADRs per patient, with prospective studies usually generating higher figures; however, it can also be assumed that no serious or otherwise significant side effects would have been missed by the physicians involved. In this survey, all available evidence is included, also taking into account congress abstracts and internal reports submitted to regulatory authorities, as they add to the overall picture and are in line with the other studies, apparently without introducing a bias. The number of ADRs is overall low, and their analysis resulted in the conclusion that they do not involve any effects potentially related to the ethanol content of the medicinal products.

The data presented here are in accordance with the conclusions drawn from data on the ADME of ethanol and the commonly accepted ethanol uptake in children with their usual nutrition [4], which is far below doses that could lead to pharmacological or toxicological effects. This also applies to the case of a study in which single doses corresponding to 142 mg ethanol were applied to children between 6 and 12 years up to 12 times per day [12], as this single dose of ethanol is eliminated within 3 min [4] so that an accumulation is not to be expected. That relevant blood ethanol levels are not achieved has recently also been confirmed by a pharmacokinetic study with one of the medicinal products included in this review, Bronchicum® Elixir [11], where children of 1–12 years of age were included. Therefore, there is no specific risk to be assigned to the ethanol content of these medicines. The ethanol content consequently does not have a relevant impact on the benefit–risk assessment of these medicines in children. This applies likewise to products with a marketing authorization based on a clinical proof of efficacy and to products registered according to EU regulations [13] based on their traditional use, as these products may also not be harmful in the specified conditions of use and their efficacy needs to be plausible on the basis of long-standing use and experience.

Therefore, the texts in the summary of the product characteristics of these products, such as those in accordance with the present regulations in Germany, which in most products only state the percentage of ethanol content in the solution [5], are appropriate, and any further texts or warnings would suggest a risk that is not relevant and could therefore be misleading.

## Conclusion

The data on pharmacokinetics, pharmacodynamics, and toxicology confirm the long-standing experience with herbal medicinal products, according to which the ethanol content of these products, such as those presently authorized or registered in EU countries, does not give cause for concern regarding their safety in children. The data do not support adding warning labels that apply to doses in the range of or even below those found in usual nutrition that is accepted as safe in children. The regulation already in force today for these products is therefore fully in accordance with the present scientific and medicinal state of the art.
